# Different Regulations of *ROM2* and *LRG1* Expression by Ccr4, Pop2, and Dhh1 in the *Saccharomyces cerevisiae* Cell Wall Integrity Pathway

**DOI:** 10.1128/mSphere.00250-16

**Published:** 2016-09-28

**Authors:** Xia Li, Tetsuro Ohmori, Kaoru Irie, Yuichi Kimura, Yasuyuki Suda, Tomoaki Mizuno, Kenji Irie

**Affiliations:** Department of Molecular Cell Biology, Graduate School of Comprehensive Human Sciences and Faculty of Medicine, University of Tsukuba, Tsukuba, Japan; Carnegie Mellon University

**Keywords:** Ccr4-Not complex, Rho1, cell wall, mRNA stability, yeasts

## Abstract

Ccr4, a component of the Ccr4-Not cytoplasmic deadenylase complex, is known to be required for the cell wall integrity (CWI) pathway in the budding yeast *Saccharomyces cerevisiae*. However, it is not fully understood how Ccr4 and other components of the Ccr4-Not complex regulate the CWI pathway. Previously, we showed that Ccr4 functions in the CWI pathway together with Khd1 RNA binding protein. Ccr4 and Khd1 modulate a signal from Rho1 small GTPase in the CWI pathway by regulating the expression of *ROM2* mRNA and *LRG1* mRNA, encoding a guanine nucleotide exchange factor (GEF) and a GTPase-activating protein (GAP) for Rho1, respectively. Here we examined the possible involvement of the *POP2* gene encoding a subunit of the Ccr4-Not complex and the *DHH1* gene encoding a DEAD box RNA helicase that associates with the Ccr4-Not complex in the regulation of *ROM2* and *LRG1* expression. Neither *ROM2* mRNA level nor Rom2 function was impaired by *pop2Δ* or *dhh1Δ* mutation. The *LRG1* mRNA level was increased in *pop2Δ* and *dhh1Δ* mutants, as well as the *ccr4Δ* mutant, and the growth defects caused by *pop2Δ* and *dhh1Δ* mutations were suppressed by *lrg1Δ* mutation. Our results suggest that *LRG1* expression is regulated by Ccr4 together with Pop2 and Dhh1 and that *ROM2* expression is regulated by Khd1 and Ccr4, but not by Pop2 and Dhh1. Thus, Rho1 activity in the CWI pathway is precisely controlled by modulation of the mRNA levels for Rho1-GEF Rom2 and Rho1-GAP Lrg1.

**IMPORTANCE** We find here that Ccr4, Pop2, and Dhh1 modulate the levels of mRNAs for specific Rho1 regulators, Rom2 and Lrg1. In budding yeast, Rho1 activity is tightly regulated both temporally and spatially. It is anticipated that Ccr4, Pop2, and Dhh1 may contribute to the precise spatiotemporal control of Rho1 activity by regulating expression of its regulators temporally and spatially. Our finding on the roles of the components of the Ccr4-Not complex in yeast would give important information for understanding the roles of the evolutionary conserved Ccr4-Not complex.

## Abstract (precis)

We find here that Ccr4, Pop2, and Dhh1 modulate the levels of mRNAs for specific Rho1 regulators, Rom2 and Lrg1. In budding yeast, Rho1 activity is tightly regulated both temporally and spatially. It is anticipated that Ccr4, Pop2, and Dhh1 may contribute to the precise spatiotemporal control of Rho1 activity by regulating expression of its regulators temporally and spatially. Our finding on the roles of the components of the Ccr4-Not complex in yeast would give important information for understanding the roles of the evolutionary conserved Ccr4-Not complex.

## INTRODUCTION

Gene expression can be regulated at many of the steps in the pathway from DNA to protein. In these regulations, posttranscriptional regulation includes the control of mRNA degradation and translation. Both 5′-cap and 3′ poly(A) tail structures of mRNAs have important roles in the control of mRNA degradation and translation. In eukaryotes, there are two general mechanisms of cytoplasmic degradation of mRNAs, 5′-to-3′ degradation and 3′-to-5′ degradation (1). Both degradations are initiated by shortening of the 3′ poly(A) tail in a process referred to as deadenylation. This deadenylation is carried out by the Pan2-Pan3 complex as well as by the Ccr4-Not complex. In the 5′-to-3′ degradation pathway, the deadenylated mRNAs are decapped by the Dcp1/Dcp2 decapping enzyme and then subjected to 5′-to-3′ degradation by Xrn1 exonuclease. Several decapping activators, such as Dhh1, Pat1, Edc3, and Scd6, stimulate the activity of decapping enzyme. In the 3′-to-5′ degradation pathway, the deadenylated mRNAs are subjected to 3′-to-5′ degradation by the exosome complex. Translation initiation is promoted by binding of the translation initiation complex eIF4F (eukaryotic initiation factor 4F) to the 5′-cap structure. This eIF4F complex contains eIF4E that directly binds to the 5′-cap structure, eIF4A that acts as an RNA helicase, and eIF4G that serves as a scaffold for the complex. Binding of the eIF4F complex to the 5′-cap structure recruits the 43S preinitiation complex, which includes the small ribosomal subunit, the initiator tRNA, and additional initiation factors (2). Translation initiation is also enhanced by the 3′ poly(A) tail and the poly(A) binding protein that interacts with eIF4G. In most cases, control of mRNA degradation and translational initiation is mediated by the 3′ untranslated regions (3′ UTR) of the regulated mRNAs where RNA binding proteins such as Puf family RNA binding proteins bind (3, 4).

The Ccr4-Not complex consists of nine core subunits, Ccr4, Pop2/Caf1, Not1, Not2, Not3, Not4, Not5, Caf40, and Caf130, in the budding yeast *Saccharomyces cerevisiae* (5, 6). In this complex, Ccr4 and Pop2 are catalytic subunits of deadenylase, and Not4 acts as a ubiquitin ligase. The *ccr4Δ* mutant shows pleiotropic phenotypes, including weak cell lysis, abnormal morphology, and defects in checkpoint control and cell cycle progression (7–11). The *pop2Δ* mutant also shows similar pleiotropic phenotypes, including weak cell lysis (7). Ccr4 and Pop2 physically and genetically interact with Dhh1, a DExD/H box protein known as decapping activator (1, 7). Overexpression of Dhh1 suppresses the phenotypes associated with *ccr4Δ* and *pop2Δ* mutant cells, and the *dhh1Δ* mutant shows a weak cell lysis phenotype, similar to *ccr4Δ* and *pop2Δ* mutants (7).

The cell wall of the budding yeast is required to maintain cell shape and integrity (12). Yeast cells must remodel the rigid structure of the cell wall during vegetative growth and during pheromone-induced morphogenesis. The cell wall remodeling is monitored and regulated by the cell wall integrity (CWI) signaling pathway (12). In the CWI signaling pathway, signals are initiated at the plasma membrane through the cell surface sensors, Wsc1, Wsc2, Wsc3, Mid2, and Mtl1. Together with phosphatidylinositol 4,5-bisphosphate (PI4,5P_2_), which recruits Rom1/2 guanine nucleotide exchange factors (GEFs) to the plasma membrane, the cell wall sensors stimulate nucleotide exchange on a small GTPase Rho1 through the activation of Rom1/2. The activated Rho1, Rho1-GTP, then activates several effectors, including protein kinase C (Pkc1), β1,3-glucan synthase, Bni1 formin protein, exocyst component Sec3, and Skn7 transcription factor. Pkc1 activates downstream mitogen-activated protein (MAP) kinase cascade, which is comprised of Bck1, Mkk1/2, and Mpk1. Mpk1 phosphorylates and activates two transcription factors, Rlm1 and the SBF complex (Swi4/Swi6), which induce gene expression. Rho1-GTP is inactivated by GTPase-activating proteins (GAPs), including Bem2, Sac7, Bag7, and Lrg1.

We have previously found that Ccr4 negatively regulates expression of the *LRG1* mRNA encoding one of the Rho1-GAPs in the CWI pathway (11). Loss of *LRG1* suppressed the cell lysis of the *ccr4Δ* mutant. Ccr4, together with RNA binding protein Khd1, also positively regulates expression of *ROM2* mRNA encoding Rho1-GEF (11). The *ccr4Δ khd1Δ* double mutant shows more severe cell lysis.

In this study, we examined the roles of Pop2 and Dhh1 in the CWI signaling pathway. The *LRG1* mRNA level was increased in *pop2Δ* and *dhh1Δ* mutants as well as *ccr4Δ* mutant and the increased *LRG1* mRNA level contributes to the growth defect of *pop2Δ* and *dhh1Δ* mutants. On the other hand, *ROM2* expression or Rom2 function was not impaired in *pop2Δ* and *dhh1Δ* mutants. Our results indicate that, in addition to the involvement of Ccr4 in the CWI signaling pathway, Dhh1 and Pop2 take a part in the regulation of Rho1 activity through the Rho1-GAP Lrg1.

## RESULTS

### The *ccr4Δ* and *pop2Δ* mutants, but not the *dhh1Δ* mutant, display a synthetic growth defect with the *khd1Δ* mutation.

**FIG 1:**
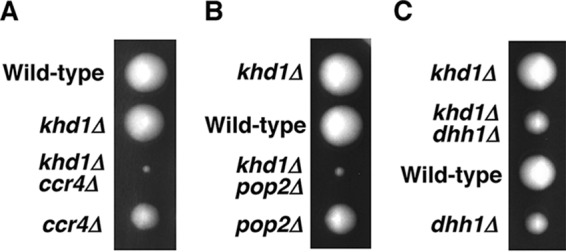
Growth of the *khd1Δ ccr4Δ*, *khd1Δ pop2Δ*, and *khd1Δ dhh1Δ* mutant strains. (A) Strain 10BD-c163 that was heterozygous for *khd1Δ* and *ccr4Δ* alleles was sporulated, and tetrads were dissected onto yeast extract-peptone-dextrose (YPD) plates. Growth after 4 days at 25°C is shown. The genotypes are indicated to the left of the image. More than 50 tetrads were dissected, and representative data are shown. (B) Strain 10BD-p163 that was heterozygous for *khd1Δ* and *pop2Δ* alleles was sporulated, and tetrads were dissected onto YPD plates. The genotypes are indicated to the left of the image. (C) Strain 10BD-d163 that was heterozygous for *khd1Δ* and *dhh1Δ* alleles was sporulated, and tetrads were dissected onto YPD plates. The genotypes are indicated to the left of the image.

We have shown that *ccr4Δ* and *pop2Δ* mutants displayed a synthetic growth defect with the *khd1Δ* mutation (11). Tetrad analysis revealed that *ccr4Δ* and *pop2Δ* mutant cells grew slower than wild-type cells, while *khd1Δ ccr4Δ* and *khd1Δ pop2Δ* double mutant cells grew much more slowly than either *khd1Δ*, *ccr4Δ*, or *pop2Δ* single mutant cells (Fig. 1A and B). To examine whether the *dhh1Δ* mutant shows a synthetic growth defect with the *khd1Δ* mutation, we performed tetrad analysis using a diploid strain that was heterozygous for *khd1Δ* and *dhh1Δ* alleles. The *dhh1Δ* mutant cells grew slower than wild-type cells, and *khd1Δ dhh1Δ* double mutant cells and *dhh1Δ* single mutant cells grew similarly (Fig. 1C). Therefore, unlike *ccr4Δ* and *pop2Δ* mutants, the *dhh1Δ* mutant does not display a synthetic growth defect with the *khd1Δ* mutation.

### *ROM2* mRNA level was not decreased in the *pop2Δ* and *dhh1Δ* mutants.

**FIG 2:**
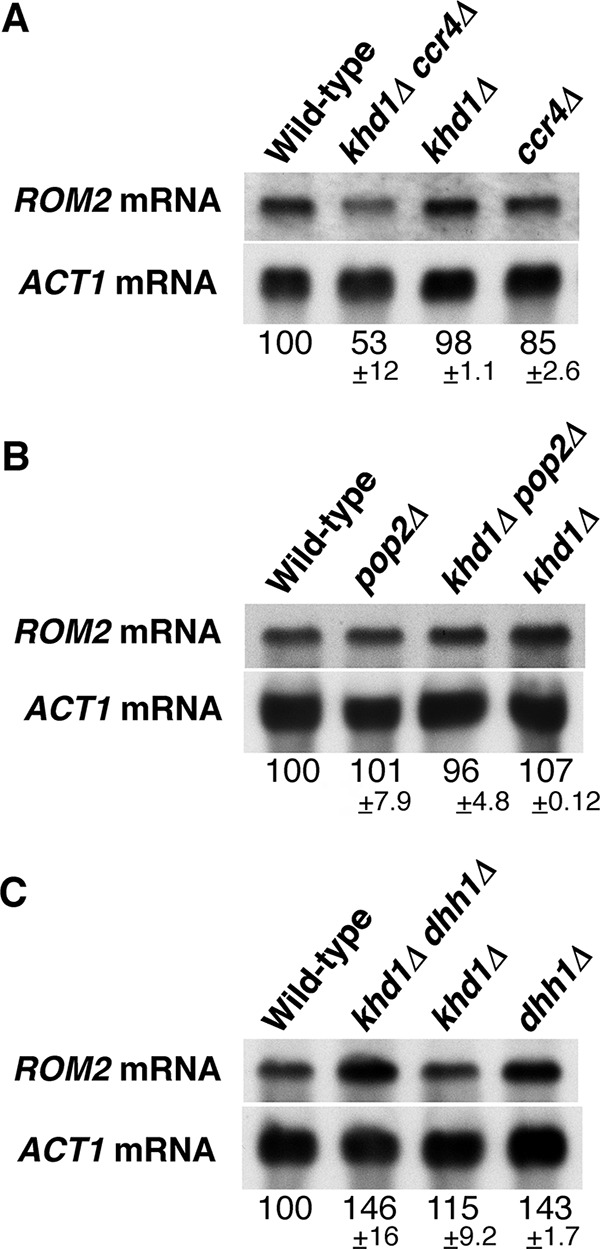
ROM2 mRNA levels in the *khd1Δ ccr4Δ*, *khd1Δ pop2Δ*, and *khd1Δ dhh1Δ* mutant strains. (A) *ROM2* mRNA levels in wild-type, *khd1Δ ccr4Δ*, *khd1Δ*, and *ccr4Δ* cells. Wild-type (c1H-1A), *khd1Δ ccr4Δ* (c1H-1B), *khd1Δ* (c1H-1C), and *ccr4Δ* (c1H-1D) cells were cultured to mid-logarithmic phase in YPD medium and collected, and total RNA was prepared. The *ROM2* transcripts were quantified by Northern blotting as described in Materials and Methods. *ACT1* mRNA was included as a quantity control. mRNA levels are shown below the lanes. The mRNA levels are shown as percentages of the wild-type levels and represent the means ± standard deviations from three independent experiments. (B) *ROM2* mRNA levels in wild-type, *pop2Δ*, *khd1Δ pop2Δ*, and *khd1Δ* cells. Wild-type (p1H-2A), *pop2Δ* (p1H-2B), *khd1Δ pop2Δ* (p1H-2C), and *khd1Δ* (p1H-2D) cells were cultured to mid-logarithmic phase in YPD medium and collected, and total RNA was prepared. (C) *ROM2* mRNA levels in wild-type, *khd1Δ dhh1Δ*, *khd1Δ*, and *dhh1Δ* cells. Wild-type (d1H-1A), *khd1Δ dhh1Δ* (d1H-1B), *khd1Δ* (d1H-1C), and *dhh1Δ* (d1H-1D) cells were cultured to mid-logarithmic phase in YPD medium and collected, and total RNA was prepared.

**FIG 3:**
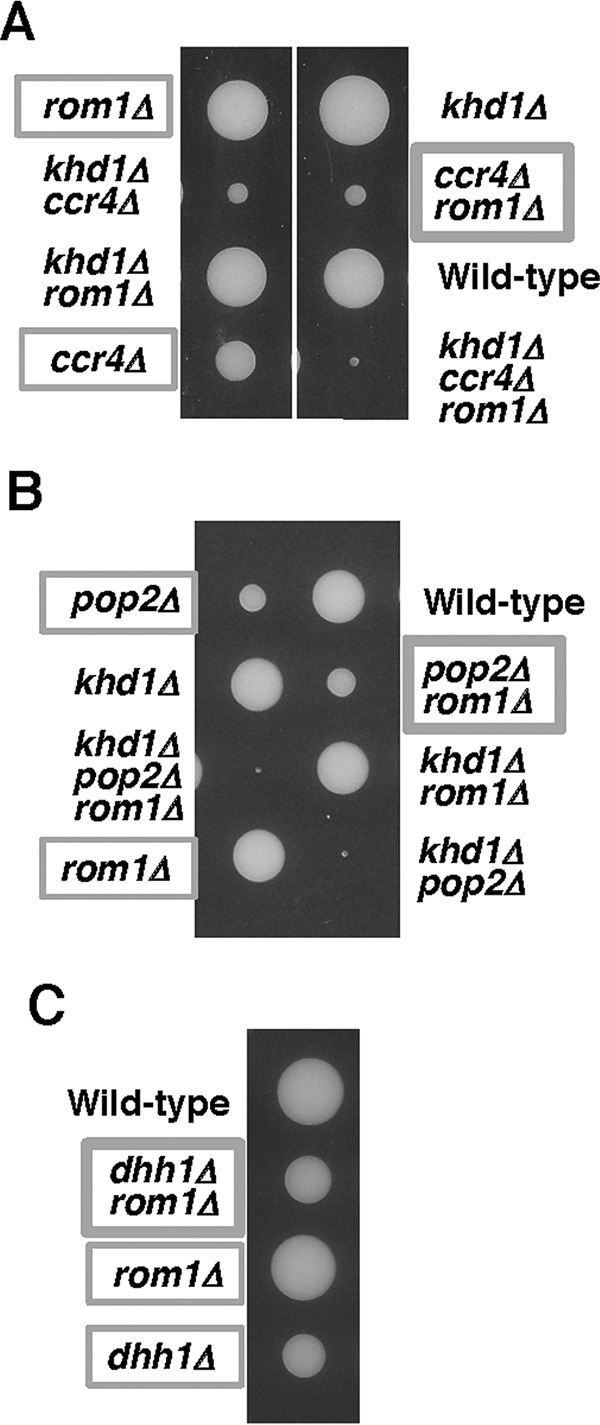
Growth of the *ccr4Δ rom1Δ*, *pop2Δ rom1*Δ, and *dhh1Δ rom1Δ* mutant strains. (A) Strain 10BD-c163r1 that was heterozygous for *khd1Δ*, *ccr4Δ*, and *rom1Δ* alleles was sporulated, and tetrads were dissected onto YPD containing 10% sorbitol. Growth after 6 days at 25°C is shown. Genotypes are indicated on both sides of the blots. More than 20 tetrads were dissected, and representative data are shown. (B) Strain 10BD-p163r1 that was heterozygous for *khd1Δ*, *pop2Δ*, and *rom1Δ* alleles was sporulated, and tetrads were dissected onto YPD containing 10% sorbitol. Growth after 6 days at 25°C is shown. Genotypes are indicated on both sides of the blots. More than 20 tetrads were dissected, and representative data are shown. (C) Strain 10BD-d1r1 that was heterozygous for *dhh1Δ* and *rom1Δ* alleles was sporulated, and tetrads were dissected onto YPD containing 10% sorbitol. Growth after 6 days at 25°C is shown. Genotypes are indicated to the left of the image. More than 20 tetrads were dissected, and representative data are shown.

We have previously shown that the level of *ROM2* mRNA (encodes Rho1 GEF) was slightly decreased in the *ccr4Δ* mutant, and this reduction was enhanced by the *khd1Δ* mutation (11) (Fig. 2A). Rom2 and Rom1 comprise a redundant pair of GEF for Rho1 (13). Loss of *ROM2* function results in temperature-sensitive growth, whereas loss of both *ROM2* and *ROM1* is lethal. Using a mutation of *ROM1*, we have obtained the genetic evidence indicating that Rom2 function was indeed impaired in *ccr4Δ* mutant and *khd1Δ ccr4Δ* double mutant cells (11) (Fig. 3A). If *ROM2* function were impaired in a strain harboring a given mutation, the mutant would show a synthetic growth defect with the *rom1Δ* mutation. Consistent with the fact that the *ROM2* mRNA level is decreased in *ccr4Δ* mutant and *khd1Δ ccr4Δ* double mutant cells, *ccr4Δ rom1Δ* double mutant cells showed much slower growth than *ccr4Δ* single mutant cells, and *khd1Δ ccr4Δ rom1Δ* triple mutant cells showed much slower growth than *khd1Δ ccr4Δ* double mutant cells (Fig. 3A). This is also consistent with the observation that overexpression of *ROM2* from a multicopy plasmid can suppress the growth defects of *khd1Δ ccr4Δ* double mutant, *ccr4Δ rom1Δ* double mutant, and *khd1Δ ccr4Δ rom1Δ* triple mutant cells (11) (see Fig. 12A) (data not shown).

We next applied this approach to examine whether the Rom2 function is impaired in *pop2Δ* and *dhh1Δ* mutants. Tetrad analysis using the diploid strain that was heterozygous for *pop2*Δ, *rom1*Δ, and *khd1Δ* alleles showed that *pop2Δ rom1Δ* double mutant cells and *pop2Δ* single mutant cells grew similarly (Fig. 3B). The *khd1Δ pop2Δ rom1Δ* triple mutant cells and *khd1Δ pop2Δ* double mutant cells also grew similarly (Fig. 3B). Tetrad analysis using the diploid strain that was heterozygous for *dhh1Δ* and *rom1Δ* alleles showed that *dhh1Δ rom1Δ* double mutant cells and *dhh1Δ* single mutant cells also grew similarly (Fig. 3C). These results suggest that Rom2 normally operates in *pop2Δ* and *dhh1Δ* mutant cells. The *ROM2* mRNA level was consistently not altered in *pop2Δ* and *khd1Δ pop2Δ* mutant cells compared to wild-type cells (Fig. 2B). Rather, *ROM2* mRNA level was marginally increased in *dhh1Δ* single mutant and *khd1Δ dhh1Δ* double mutant cells (Fig. 2C). Thus, Rom2 function and *ROM2* expression were impaired in *ccr4Δ* mutant and *khd1Δ ccr4Δ* double mutant cells, but not in *pop2Δ* and *dhh1Δ* mutant cells. These results indicate that only Ccr4 functions in regulation of the expression level of *ROM2* mRNA.

### Rom2 protein level was decreased in *ccr4*Δ and *khd1*Δ *ccr4*Δ mutants.

**FIG 4:**
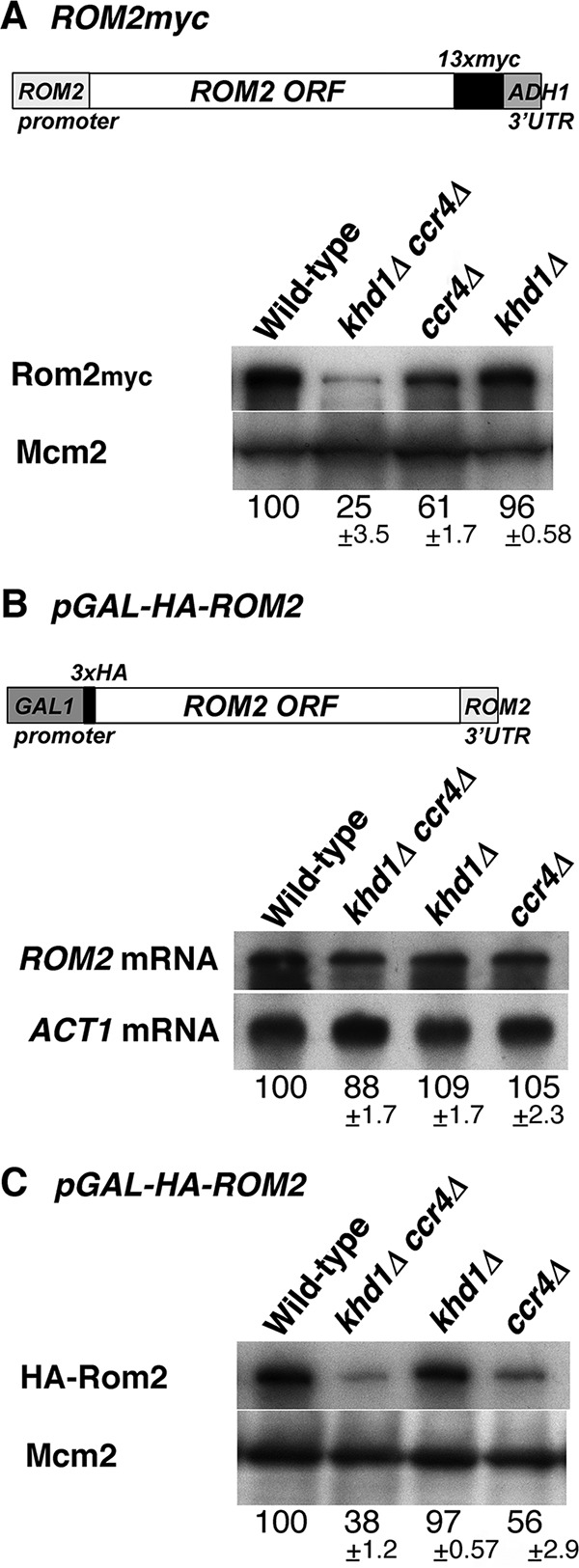
Rom2 protein levels in the *ccr4Δ* and *khd1Δ ccr4Δ* mutant strains. (A, top) Schematic representation of the regulatory elements in the *ROM2myc* construct. (Bottom) Rom2myc protein levels in wild-type, *khd1Δ ccr4Δ*, *khd1Δ*, and *ccr4Δ* cells. Wild-type (180-3B-4A), *khd1Δ ccr4Δ* (180-3B-1A), *ccr4Δ* (180-3B-7C), and *khd1Δ* (180-3B-7D) cells harboring the *ROM2myc* construct were cultured to mid-logarithmic phase in YPD medium and collected, and total protein was prepared. The Rom2myc proteins were quantified by Western blotting as described in Materials and Methods. Mcm2 protein was included as a quantity control. The protein levels shown below the lanes are indicated as percentages of the wild-type levels and represent the means ± standard deviations from three independent experiments. (B, top) Schematic representation of the regulatory elements in the pGAL-HA-ROM2 construct. (Bottom) *HA-ROM2* mRNA levels in wild-type, *khd1Δ ccr4Δ*, *khd1Δ*, and *ccr4Δ* cells. Wild-type (c1H-1A), *khd1Δ ccr4Δ* (c1H-1B), *khd1Δ* (c1H-1C), and *ccr4Δ* (c1H-1D) cells harboring pGAL-HA-ROM2 plasmid were cultured to mid-logarithmic phase in SG−Ura medium and collected, and total RNA was prepared. The *HA-ROM2* transcripts were quantified by Northern blotting as described in Materials and Methods. *ACT1* mRNA was included as a quantity control. The mRNA levels are indicated as percentages of wild-type levels and represent the means ± standard deviations from three independent experiments. (C) HA-Rom2 protein levels in wild-type, *khd1Δ ccr4Δ*, *khd1Δ*, and *ccr4Δ* cells. Wild-type (c1H-1A), *khd1Δ ccr4Δ* (c1H-1B), *khd1Δ* (c1H-1C), and *ccr4Δ* (c1H-1D) cells harboring pGAL-HA-ROM2 plasmid were cultured to mid-logarithmic phase in SG−Ura medium and collected, and total protein was prepared. The HA-Rom2 proteins were quantified by Western blotting as described in Materials and Methods. Mcm2 protein was included as a quantity control. The protein levels are indicated as percentages of wild-type levels and represent the means ± standard deviations from three independent experiments.

To address how Ccr4 functions in *ROM2* expression, we quantified the level of Rom2 protein in *ccr4Δ* single mutant and *khd1Δ ccr4Δ* double mutant cells using the myc-tagged *ROM2* construct. As shown in Fig. 4A, myc-tagged Rom2 (Rom2myc) protein levels were decreased in *ccr4Δ* single mutant and *khd1Δ ccr4Δ* double mutant cells compared to wild-type cells. Decreased protein levels in *ccr4Δ* and *khd1Δ ccr4Δ* mutant cells (61% in *ccr4Δ* mutant and 25% in *khd1Δ ccr4Δ* mutant cells in Fig. 4A) were more evident than the decreased mRNA levels (85% in *ccr4Δ* mutant and 53% in *khd1Δ ccr4Δ* mutant cells in Fig. 2A), implying that Rom2 expression is regulated at both mRNA and protein levels. The myc-tagged *ROM2* construct used here had the *ADH1* 3′ UTR instead of endogenous *ROM2* 3′ UTR (Fig. 4A), implying that the *ROM2* 3′ UTR is not essential for the regulation of *ROM2* expression. To investigate the protein level regulation, we utilized the pGAL-HA-ROM2 construct harboring the *ROM2* 3′ UTR (Fig. 4B and C). While the *HA-ROM2* mRNA levels from the *GAL1* promoter were not altered in *ccr4Δ* and *khd1Δ ccr4Δ* mutant cells compared to wild-type cells (Fig. 4B), the hemagglutinin-tagged Rom2 (HA-Rom2) protein levels were clearly decreased in *ccr4Δ* and *khd1Δ ccr4Δ* mutant cells compared to wild-type cells (Fig. 4C). Together with the observation that *ROM2* mRNA levels from the endogenous *ROM2* promoter were slightly decreased in *ccr4Δ* mutant and *khd1Δ ccr4Δ* double mutant cells (Fig. 2A), Rom2 expression is likely to be regulated at the both mRNA and protein levels.

**FIG 5:**
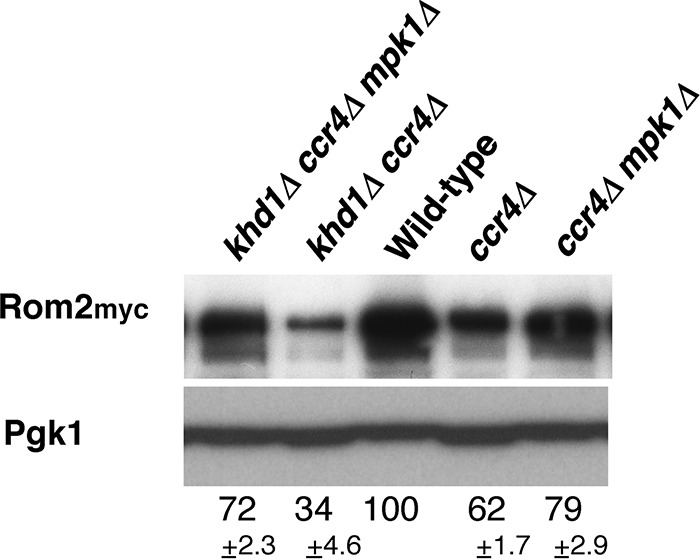
Rom2 protein levels in the *ccr4Δ*, *khd1Δ ccr4Δ*, *ccr4Δ mpk1*Δ, and *khd1Δ ccr4Δ mpk1Δ* mutant strains. The Rom2myc protein levels in wild-type, *ccr4Δ*, *khd1Δ ccr4Δ*, *ccr4Δ* *mpk1Δ*, and *ccr4Δ khd1Δ mpk1Δ* cells are shown. Wild-type (180-m-1D4A), *khd1Δ ccr4Δ* (180-m-3D), *khd1Δ ccr4Δ mpk1Δ* (180-m-7A), *ccr4Δ* (180-m-6B), and *ccr4Δ mpk1Δ* (180-m-4C) cells harboring the *ROM2myc* construct were cultured to mid-logarithmic phase in YPD containing 10% sorbitol medium and collected, and total protein was prepared. The Rom2myc proteins were quantified by Western blotting as described in Materials and Methods. Pgk1 protein was included as a quantity control. The protein levels are indicated as percentages of wild-type levels and represent the means ± standard deviations from three independent experiments.

We have previously shown that the *ccr4Δ* single mutant shows weak cell lysis and that the *khd1Δ ccr4Δ* double mutant shows more severe cell lysis (11). Due to the cell lysis, Mpk1 is constitutively activated in *ccr4Δ* and *khd1Δ ccr4Δ* mutants (data not shown). Since it has been reported that Mpk1 downregulates Rom2 (14), we speculated that Mpk1 might be involved in *ROM2* expression. We found that the decreased Rom2myc protein levels in *ccr4Δ* and *khd1Δ ccr4Δ* mutants were partially suppressed by the *mpk1Δ* mutation (Fig. 5). Thus, the decreased Rom2myc protein levels in *ccr4Δ* and *khd1Δ ccr4Δ* mutants are partly due to the constitutive activation of Mpk1.

### *LRG1* expression is negatively regulated by Pop2 and Dhh1.

**FIG 6:**
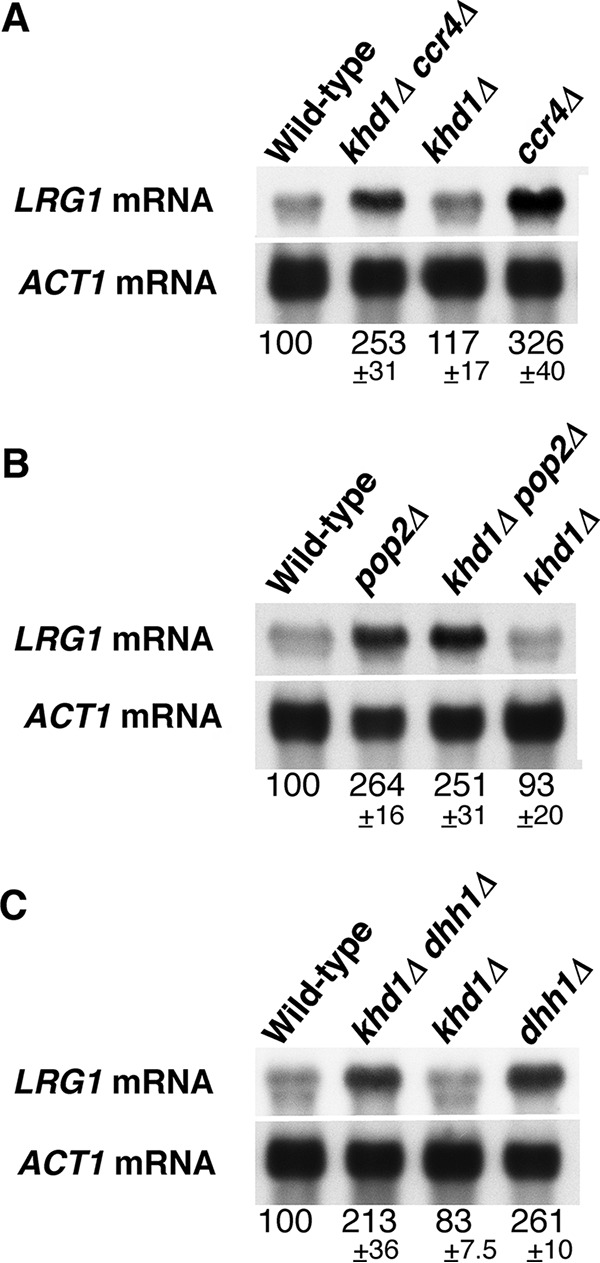
LRG1 mRNA levels in the *khd1Δ ccr4Δ*, *khd1Δ pop2Δ*, and *khd1Δ dhh1Δ* mutant strains. (A) *LRG1* mRNA levels in wild-type, *khd1Δ ccr4Δ*, *khd1Δ*, and *ccr4Δ* cells. Wild-type (c1H-1A), *khd1Δ ccr4Δ* (c1H-1B), *khd1Δ* (c1H-1C), and *ccr4Δ* (c1H-1D) cells were cultured to mid-logarithmic phase in YPD medium and collected, and total RNA was prepared. The *LRG1* transcripts were quantified by Northern blotting as described in Materials and Methods. *ACT1* mRNA was included as a quantity control. The mRNA levels are indicated as percentages of wild-type levels and represent the means ± standard deviations from three independent experiments. The bands smaller than the *LRG1* mRNA bands show cross hybridization to rRNA. (B) *LRG1* mRNA levels in wild-type, *pop2Δ*, *khd1Δ pop2Δ*, and *khd1Δ* cells. Wild-type (p1H-2A), *pop2Δ* (p1H-2B), *khd1Δ pop2Δ* (p1H-2C), and *khd1Δ* (p1H-2D) cells were cultured to mid-logarithmic phase in YPD medium and collected, and total RNA was prepared. (C) *LRG1* mRNA levels in wild-type, *khd1Δ dhh1Δ*, *khd1Δ*, and *dhh1Δ* cells. Wild-type (d1H-1A), *khd1Δ dhh1Δ* (d1H-1B), *khd1Δ* (d1H-1C), and *dhh1Δ* (d1H-1D) cells were cultured to mid-logarithmic phase in YPD medium and collected, and total RNA was prepared.

We have previously shown that the level of *LRG1* mRNA encoding Rho1 GAP was increased in the *ccr4Δ* single mutant and *khd1Δ ccr4Δ* double mutant cells (11) (Fig. 6A). Therefore, we quantified *LRG1* mRNA levels in *pop2Δ* single mutant and *khd1Δ pop2Δ* double mutant cells. As shown in Fig. 6B, *LRG1* mRNA levels were increased in *pop2Δ* and *khd1Δ pop2Δ* mutant cells than in wild-type cells. In addition, we found that *LRG1* mRNA levels were increased in *dhh1Δ* and *khd1Δ dhh1Δ* mutant cells than in wild-type cells (Fig. 6C). Therefore, *LRG1* expression is downregulated by Pop2 and Dhh1.

**FIG 7:**
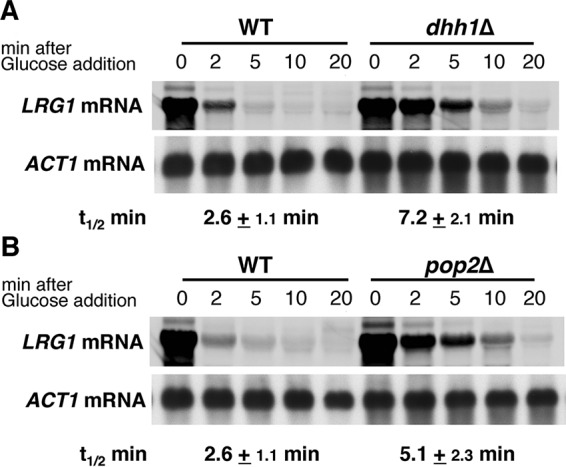
Degradation of the *LRG1* mRNA in the *pop2Δ* and *dhh1Δ* mutant strains. (A) Wild-type (WT) d1H-1A cells carrying the pGAL-LRG1 plasmid and *dhh1*Δ cells (d1H-1D cells) carrying pGAL-LRG1 plasmid. (B) WT cells (p1H-2A) carrying pGAL-LRG1 plasmid and *pop2*Δ cells (p1H-2B) carrying pGAL-LRG1 plasmid. Cells harboring the pGAL-LRG1 plasmid were grown in SG−Ura, and the medium was changed to SC−Ura to inhibit transcription from the *GAL1* promoter. Cells were harvested at the times indicated above the lanes, and total RNA was isolated. Samples were analyzed by Northern blotting with specific probes, and the half-lives (*t*_1/2_) (in minutes) were determined as the means from three independent experiments. *ACT1* mRNA was used as a reference for quantification.

Pop2 and Dhh1 encode a cytoplasmic deadenylase and a DExD/H box RNA helicase known as mRNA decapping activator, respectively, and they are important factors acting in mRNA degradation (1). Therefore, we speculate that Pop2 and Dhh1 are involved in the degradation of *LRG1* mRNA. To analyze the decay rates of *LRG1* mRNA, we employed the controllable *GAL1* promoter to express *LRG1* mRNA. As shown in Fig. 7A and B, *LRG1* mRNA were stabilized in *pop2Δ* and *dhh1Δ* mutant cells. Notably, in *pop2Δ* and *dhh1Δ* mutant cells, *LRG1* mRNA has a twofold-longer half-life than in wild-type cells. These results indicate that Pop2 and Dhh1 are involved in the degradation of *LRG1* mRNA.

### Loss of *LRG1* suppresses the growth defect of the *pop2Δ* and *dhh1Δ* mutations.

**FIG 8:**
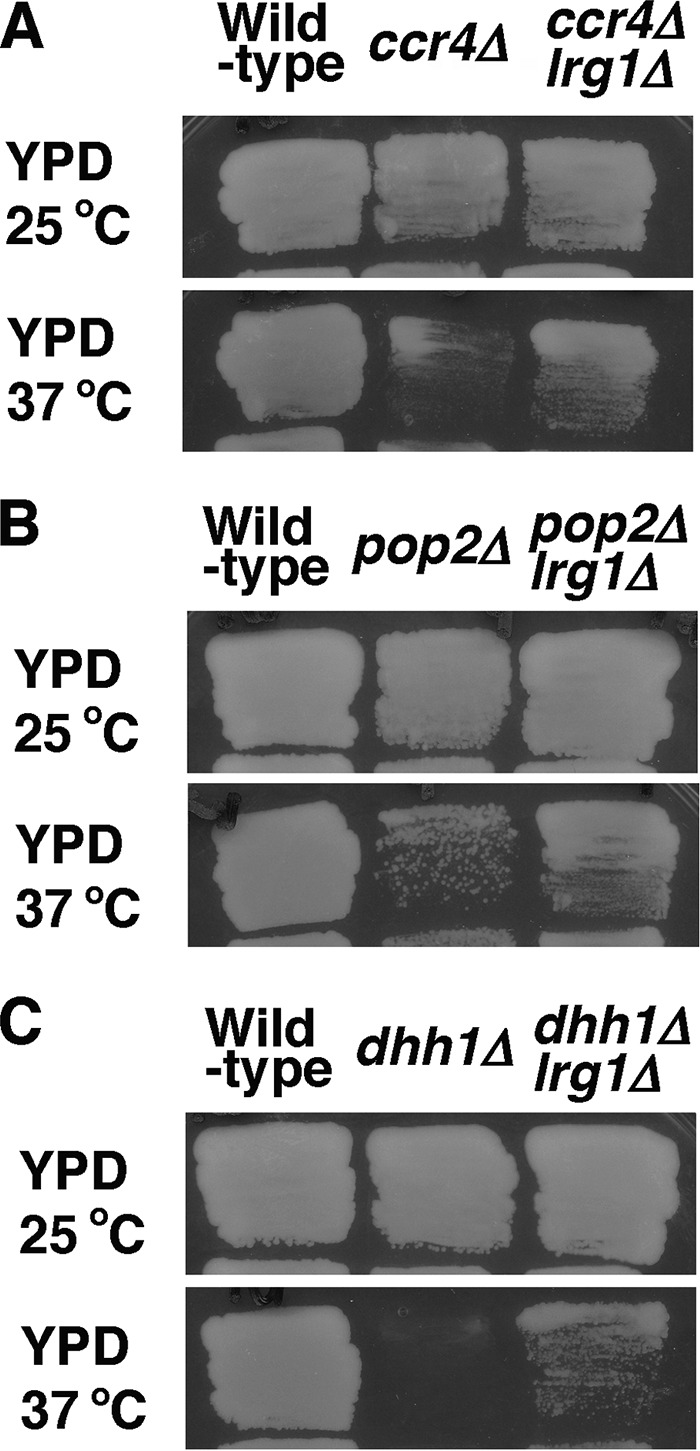
Loss of *LRG1* suppresses cell lysis of the *ccr4Δ*, *pop2Δ*, and *dhh1Δ* mutants. (A) Wild-type (c1H-1A), *ccr4Δ* (c1H-1D), and *ccr4Δ lrg1Δ* (cl4-1B) cells were plated on YPD medium plates and grown at either 25°C or 37°C for 3 days. (B) Wild-type (p1H-2A), *pop2Δ* (p1H-2B), and *pop2Δ lrg1Δ* (pl4-1B) cells were plated on YPD medium plates and grown at either 25°C or 37°C for 3 days. (C) Wild-type (d1H-1A), *dhh1Δ* (d1H-1D), and *dhh1Δ lrg1Δ* (dl4-1B) cells were plated on YPD medium plates and grown at either 25°C or 37°C for 3 days.

We have shown that *LRG1* mRNA expression is increased in the *khd1Δ ccr4Δ* mutant and that deletion of *LRG1* suppressed the growth defect of the *khd1Δ ccr4Δ* mutant (11). At high temperature, the severe growth defect was observed even in the *ccr4Δ* single mutant (Fig. 8A). The defect associated with the *ccr4Δ* single mutation was effectively suppressed by deletion of *LRG1* (Fig. 8A), indicating that the increased level of *LRG1* contributes to the growth defect of *ccr4Δ* mutant cells. Since *LRG1* mRNA levels were also increased in *pop2Δ* and *dhh1Δ* mutant cells, we examined whether deletion of *LRG1* can also suppress the growth defect caused by *pop2Δ* and *dhh1Δ* mutations. The *pop2Δ* and *dhh1Δ* mutant cells failed to grow at elevated temperature (37°C) (Fig. 8B and C). Their growth defects are due to cell lysis, since addition of osmotic stabilizer sorbitol to medium improved their growth at 37°C (data not shown). The *pop2Δ lrg1Δ* and *dhh1Δ lrg1Δ* double mutant cells could grow at 37°C, although their growth was slightly slower than that of wild-type cells (Fig. 8B and C). These results indicate that the increased *LRG1* mRNA level contributes to the growth defect of *pop2Δ* and *dhh1Δ* mutant cells.

**FIG 9:**
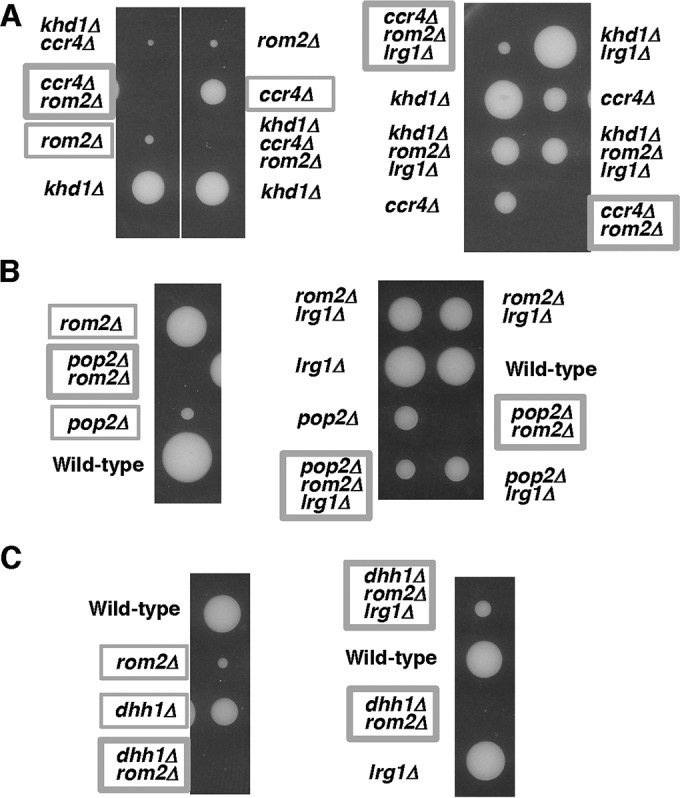
Loss of *LRG1* suppresses the lethality of the *ccr4Δ* rom2Δ, *pop2Δ rom2Δ*, and *dhh1Δ rom2Δ* mutants. (A) Strain 10BD-c163r2l1 that was heterozygous for *khd1Δ*, *ccr4Δ*, *rom2Δ*, and *lrg1Δ* alleles was sporulated, and tetrads were dissected onto YPD containing 10% sorbitol. Growth after 6 days at 25°C is shown. Genotypes are indicated on both sides of the blots. More than 20 tetrads were dissected, and representative data are shown. (B) Strain 10BD-pr1l1 that was heterozygous for *pop2Δ*, *rom2Δ*, and *lrg1Δ* alleles was sporulated, and tetrads were dissected onto YPD containing 10% sorbitol. Growth after 6 days at 25°C is shown. Genotypes are indicated on both sides. More than 20 tetrads were dissected, and representative data are shown. (C) Strain 10BD-d1r1l1 that was heterozygous for *dhh1Δ*, *rom2Δ*, and *lrg1Δ* alleles was sporulated, and tetrads were dissected onto YPD containing 10% sorbitol. Growth after 6 days at 25°C is shown. Genotypes are indicated to the left of the blots. More than 20 tetrads were dissected, and representative data are shown.

We have previously shown that *ccr4Δ rom2Δ* double mutants and *khd1Δ ccr4Δ rom2Δ* triple mutants were inviable (11) (Fig. 9A). This raised the possibility that the lethality of the *ccr4Δ rom2Δ* mutant was attributed to the increased *LRG1* mRNA level. To test this, we examined whether the *lrg1*Δ mutation suppresses the growth defect of the *ccr4Δ rom2Δ* mutant. Indeed, the *lrg1*Δ mutation suppressed the growth defect of the *ccr4Δ rom2Δ* mutant (Fig. 9A). We then examined growth of *pop2Δ rom2Δ* and *dhh1Δ rom2Δ* double mutant cells and found that both mutants were also inviable (Fig. 9B and C). The *lrg1Δ* mutation also suppressed the growth defect of *pop2Δ rom2*Δ and *dhh1Δ rom2Δ* mutants (Fig. 9B and C), indicating that the increased *LRG1* mRNA level causes the lethality in the *pop2Δ rom2*Δ and *dhh1Δ rom2*Δ mutants. These results suggest that *LRG1* mRNA is a target mRNA for Ccr4, Pop2, and Dhh1 and that regulation of the *LRG1* mRNA stability mediated by Ccr4, Pop2, and Dhh1 is important for yeast cells to grow at high temperature.

### Overexpression of Dhh1 suppressed the growth defect of the *khd1Δ ccr4Δ* mutant.

**FIG 10:**
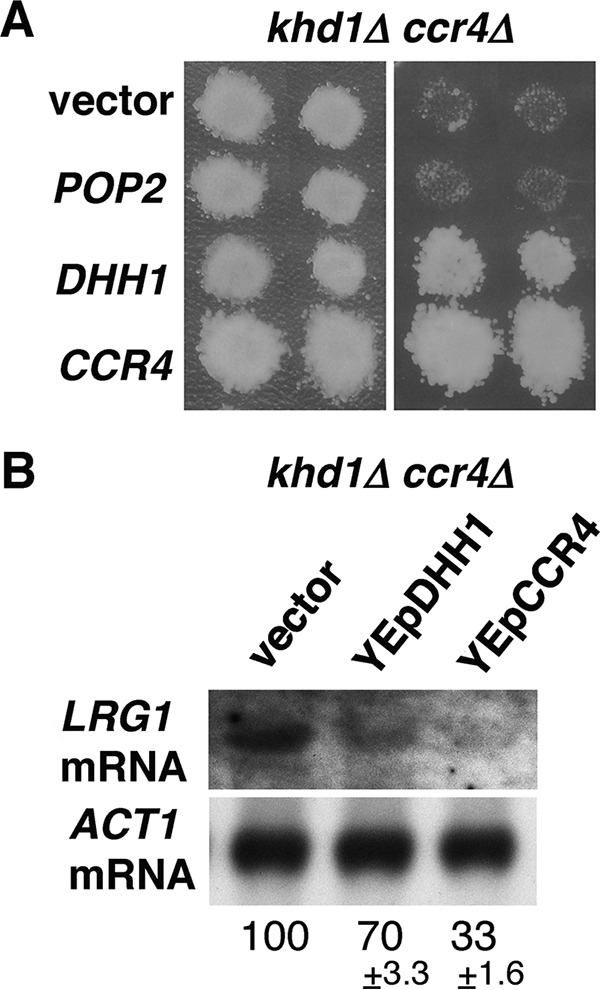
Overexpression of *DHH1* suppresses the growth defect of *khd1Δ ccr4Δ* double mutants. (A) Multicopy suppressors of *khd1Δ ccr4Δ*. Transformants of the *khd1Δ ccr4Δ* strain (c1H-1B) carrying the plasmid indicated to the left of the blot were streaked onto YPD medium and incubated at 25°C (left) or 37°C (right). Each patch represents an independent transformant. The plasmids were YEplac195 (vector), YEplac195-POP2 (*POP2*), YEplac195-DHH1 (*DHH1*), and YEplac195-CCR4 (*CCR4*). (B) *LRG1* mRNA levels in *khd1Δ ccr4Δ* cells harboring plasmid. The *khd1Δ ccr4Δ* (c1H-1B) cells harboring vector, YEplac195-DHH1, or YEplac195-CCR4 were cultured to mid-logarithmic phase in SC−Ura medium and collected, and total RNA was prepared. The *LRG1* transcripts were quantified by Northern blotting as described in Materials and Methods. *ACT1* mRNA was included as a quantity control. The mRNA levels are indicated as percentages of the cells harboring vector and represent the means ± standard deviations from two independent experiments.

A previous study showed not only that Dhh1 interacts physically with Ccr4 and Pop2 but also that overexpression of Dhh1 suppressed the phenotypes associated with the *pop2Δ* and *ccr4Δ* mutations (7). These results raised the possibility that Dhh1 overexpression could suppress the growth defect of the *khd1Δ ccr4Δ* mutant. To test this, we transformed multicopy plasmids carrying either the *DHH1*, *CCR4*, or *POP2* gene into *khd1Δ ccr4Δ* mutant cells. As shown in Fig. 10A, overexpression of Dhh1 suppressed the growth defect of the *khd1Δ ccr4Δ* mutant at 37°C, but overexpression of Pop2 did not. In the *khd1Δ ccr4Δ* mutant, the expression levels of *ROM2* and *LRG1* mRNAs are decreased and increased, respectively (11) (Fig. 2A and 6A). We hypothesized that *DHH1* overexpression suppresses the growth defect of the *khd1Δ ccr4Δ* mutant by reducing *LRG1* expression, since the *dhh1Δ* mutation affects *LRG1* expression, but not *ROM2* expression (Fig. 2C and 6C). Indeed, as shown in Fig. 10B, the *LRG1* mRNA level in the *khd1Δ ccr4Δ* mutant was reduced by Dhh1 overexpression. This result supports the model in which Dhh1 negatively regulates *LRG1* expression.

**FIG 11:**
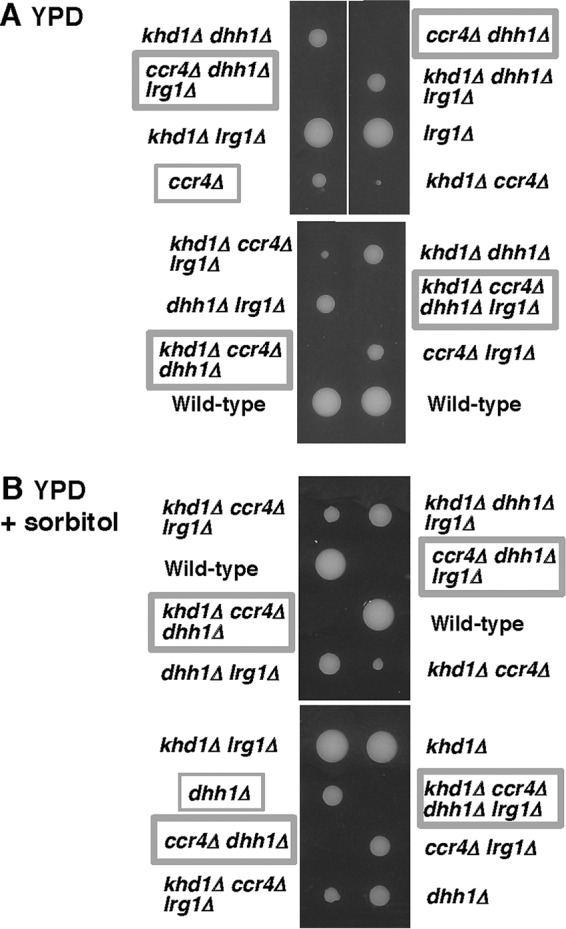
Growth of the *ccr4Δ dhh1Δ* and *ccr4Δ dhh1Δ lrg1Δ* mutant strains. Strain 10BD-c163d1l1 that was heterozygous for *khd1Δ*, *ccr4Δ*, *dhh1Δ*, and *lrg1Δ* alleles was sporulated, and tetrads were dissected onto YPD (A) and YPD containing 10% sorbitol (B). Growth after 6 days at 25°C is shown. Genotypes are indicated on both sides of the blots. More than 20 tetrads were dissected, and representative data are shown.

To clarify whether Ccr4 and Dhh1 function in the linear pathway, we examined the growth of *ccr4Δ dhh1Δ* double mutant cells. Surprisingly, *ccr4Δ dhh1Δ* double mutant cells are inviable (Fig. 11A). This result was inconsistent with a previous observation of Hata et al. (7), in which *ccr4Δ* and *dhh1Δ* mutations do not have any additive phenotypes. Deletion of *LRG1* failed to suppress the growth defect of *ccr4Δ dhh1Δ* double mutant cells (Fig. 11). Furthermore, the addition of sorbitol, an active allele of *RHO1* (Q-to-L change at position 68 encoded by *RHO1* [*RHO1-Q68L*]), or an active allele of *PKC1* (*PKC1-R398P*) failed to suppress the growth defect of the *ccr4Δ dhh1Δ* double mutant (Fig. 11B and data not shown). These results suggest that, in addition to the CWI pathway, Ccr4 and Dhh1 cooperatively regulate another biological process.

### Different roles of Ccr4 and Pop2 in the CWI pathway.

**FIG 12:**
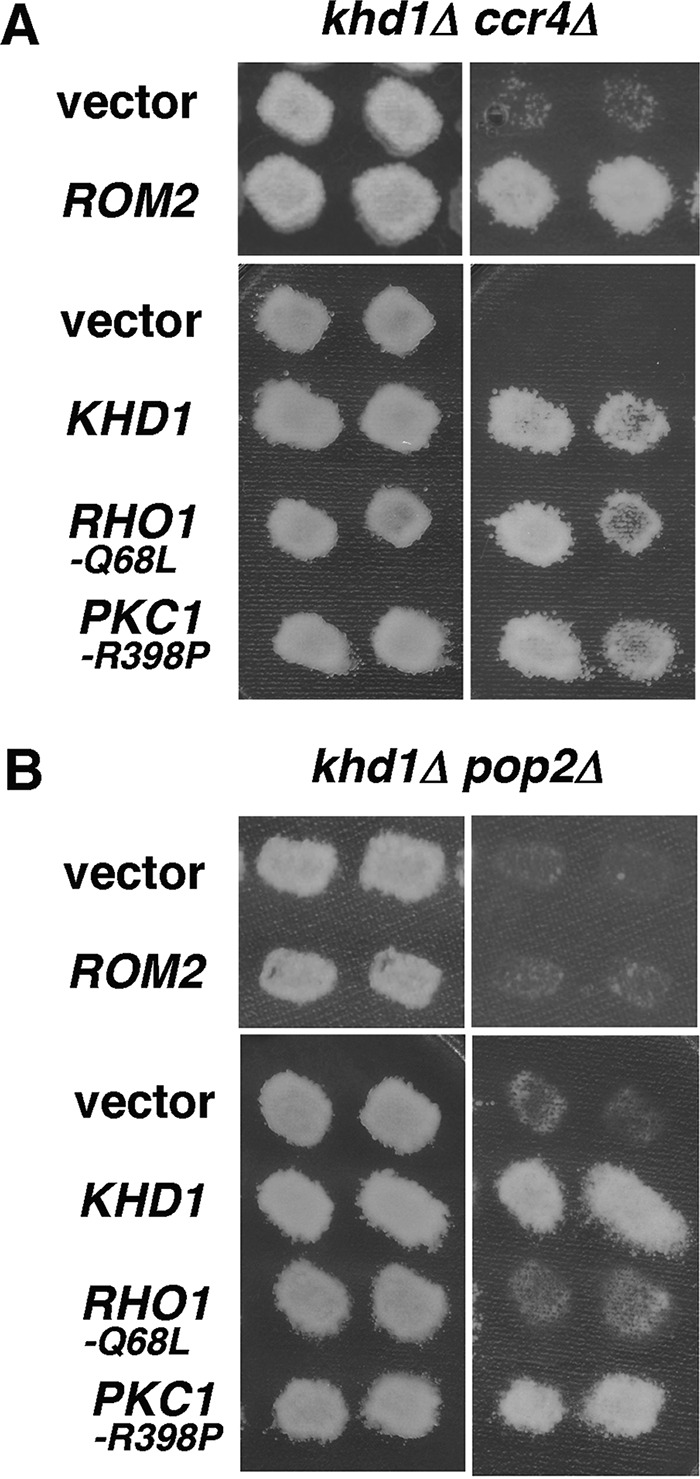
Different roles of Ccr4 and Pop2 in the CWI pathway. (A) Overexpression of *ROM2* and expression of activated Rho1 and Pkc1 alleles suppress the growth defect of *khd1Δ ccr4Δ* double mutants. The plasmids were the ROM2 plasmid and plasmid harboring activated Rho1 and Pkc1 alleles in *khd1Δ ccr4Δ*. Transformants of the *khd1Δ ccr4Δ* strain (c1H-1B) carrying the indicated plasmids were streaked onto YPD medium and incubated at 25°C (left) or 37°C (right). Each patch represents an independent transformant. Plasmids were YEplac195 (vector), YEplac195-ROM2 (*ROM2*), YCplac33 (vector), YCplac-RHO1-Q68L (*RHO1-Q68L*), and YCplac33-PKC1-R398P (*PKC1-R398P*). (B) Overexpression of *ROM2* and expression of activated Rho1 and Pkc1 alleles suppress the growth defect of *khd1Δ pop2Δ* double mutants. The plasmids were the ROM2 plasmid and plasmid harboring activated Rho1 and Pkc1 alleles in *khd1Δ pop2Δ*. Transformants of the *khd1Δ pop2Δ* strain (p1H-2C) carrying the indicated plasmids were streaked onto YPD medium and incubated at 25 °C (left) or 37°C (right).

Both *ccr4Δ* and *pop2Δ* mutants displayed a synthetic growth defect with the *khd1Δ* mutation (11). In the *khd1Δ ccr4Δ* double mutant, Rom2 function is decreased and Lrg1 function is increased. These results suggest that, in the *khd1Δ ccr4Δ* double mutant, Rho1 activity is severely decreased, which results in its growth defect. This idea is supported by the findings that the growth defect of the *khd1Δ ccr4Δ* double mutant could be suppressed by *ROM2* overexpression and expression of *RHO1-Q68L* (11) (Fig. 12A). On the other hand, in the *khd1Δ pop2Δ* double mutant, Lrg1 function is increased, but Rom2 function is normal. Therefore, it is anticipated that a reduction of Rho1 activity is less severe in *khd1Δ pop2Δ* double mutant cells than in *khd1Δ ccr4Δ* double mutant cells. *ROM2* overexpression and *RHO1-Q68L* failed to suppress the growth defect of the *khd1Δ pop2Δ* double mutant (Fig. 12B), indicating that decreased Rho1 activity caused by the increased Lrg1 level cannot account for the growth defect of the *khd1Δ pop2Δ* double mutant.

Rho1 acts as an activator of five effectors, including Pkc1, Fks1, Bni1, Sec3, and Skn7 (12). The growth defect of the *khd1Δ ccr4Δ* double mutant can be suppressed by *PKC1-R398P* (11) (Fig. 12A), suggesting that reduction of Pkc1 activity is responsible for the growth defect of the *khd1Δ ccr4Δ* double mutant. We unexpectedly found that *PKC1-R398P* also suppressed the growth defect of the *khd1Δ pop2Δ* double mutant (Fig. 12B). This result suggests that the signaling from Rho1 to Pkc1 requires a cooperative function of Pop2 and Khd1. Taken together, these results suggest that Pop2 and Ccr4 not only destabilize a common target, *LRG1* mRNA, but also function upstream of Pkc1 in the CWI pathway in a manner independent of each other.

## DISCUSSION

In this study, we found that the *LRG1* mRNA level was increased in *pop2Δ* and *dhh1Δ* mutants and the *ccr4Δ* mutant than in the wild type. The growth defect of *pop2Δ* and *dhh1Δ* mutants at high temperature and the lethality of *pop2Δ rom2Δ* and *dhh1Δ rom2* double mutants are suppressed by the *lrg1Δ* mutation. Thus, the increased *LRG1* mRNA level does contribute to the growth defect of *pop2Δ* and *dhh1Δ* mutants. The *ccr4Δ*, *pop2Δ*, and *dhh1Δ* mutants show more severe growth defects at high temperature, suggesting that the negative regulation of *LRG1* expression is more important at high temperature. Since it is well-known that the CWI pathway is activated at high temperature (12, 15), the negative regulation of *LRG1* expression is more important at high temperature to ensure the proper activation of Rho1 at high temperature. Besides Lrg1, there are three other Rho1-GAPs, Bem2, Sac7, and Bag7 (12). The level of expression of *BEM2*, *SAC7*, or *BAG7* mRNA was not altered significantly in *ccr4Δ* and *pop2Δ* mutants (data not shown). In these GAPs, Lrg1 has been reported to participate in the regulation of β-1,3-glucan synthase (16). Bem2 and Sac7 are involved in the downregulation of the Pkc1-activated mitogen-activated protein kinase (MAPK) pathway (17, 18). Thus, it is possible that the negative regulation of *LRG1* expression by Ccr4, Pop2, and Dhh1 is important for Rho1 to activate β-1,3-glucan synthase properly. Data from *Candida albicans* also support this idea, as *ccr4* and *pop2* mutants showed relatively lower glucan in the cell wall (19).

How is *LRG1* mRNA specifically recognized by Ccr4, Pop2, and Dhh1 as a target mRNA? Stewart et al. (20) have reported that an RNA binding protein Puf5/Mpt5 negatively regulates the *LRG1* mRNA level and that the *lrg1Δ* mutation suppresses the growth defect of the *puf5Δ* mutant. Puf5 was originally isolated as a multicopy suppressor of the *pop2* mutation (7). Puf5 directly binds to the 3′ UTR of *LRG1* mRNA (21, 22) and physically interacts with Ccr4, Pop2, and Dhh1 (4, 7). Previous studies showed that Puf5 does not bind to the 3′ UTR of *BEM2*, *SAC7*, or *BAG7* mRNA encoding other Rho1-GAPs (21, 22). Thus, Ccr4, Pop2, and Dhh1 may specifically regulate *LRG1* mRNA via the ability of Puf5 to recruit them to the 3′ UTR of *LRG1* mRNA.

### Ccr4 and Pop2 shorten the poly(A) tail of *LRG1* mRNA, and Dhh1 stimulates decapping by Dcp1/2.

The *ccr4*Δ, *pop2Δ*, and *dhh1Δ* mutants show severe growth defect at high temperature, and their growth defects are suppressed by *lrg1*Δ mutation, suggesting that rapid degradation of *LRG1* mRNA is important for cell growth, especially at high temperature. We have shown here that overexpression of Dhh1 suppressed the growth defect of the *khd1Δ ccr4Δ* mutant at 37°C and that the elevated *LRG1* mRNA level in the *khd1Δ ccr4Δ* mutant was reduced by Dhh1 overexpression. Dhh1 overexpression could be inducing the deadenylation-independent decapping, and in that way confer a decrease of the *LRG1* mRNA level. Thus, Dhh1 acts downstream of Ccr4 in the degradation pathway of *LRG1* mRNA. While Ccr4, Pop2, and Dhh1 share *LRG1* mRNA as a target, they may act independently on other targets. We found here that the combination of *ccr4Δ dhh1Δ* mutations was lethal and that deletion of *LRG1* failed to suppress the lethality of the *ccr4Δ dhh1Δ* double mutant. Thus, the *LRG1* mRNA is not the sole target mRNA for Ccr4 and Dhh1. Since Ccr4 and Dhh1 are global regulators acting on practically all mRNAs, the lethality of *ccr4*Δ *dhh1*Δ double mutant cells could be caused by more general changes in mRNA degradation and translational repression, rather than control of specific target mRNAs.

The level of *ROM2* mRNA encoding Rho1 GEF was slightly decreased in the *ccr4Δ* mutant, and this reduction was enhanced by the *khd1Δ* mutation (11) (Fig. 2A). We also confirmed genetically that Rom2 function was indeed impaired in the *ccr4Δ* single mutant and *khd1Δ ccr4Δ* double mutant using a mutation of the *ROM1* gene. Using this genetic approach, we found that Rom2 function was not impaired in *pop2Δ* and *dhh1Δ* mutants. Consistently, *ROM2* mRNA level was not decreased in *pop2Δ* and *dhh1Δ* mutants. Thus, Ccr4 acts independently of Pop2 and Dhh1 in regulating *ROM2* expression. Rom2 protein levels and *ROM2* mRNA levels were decreased in *ccr4Δ* and *khd1Δ ccr4Δ* mutants than in wild-type cells, and the decreased protein levels were more evident than the decreased mRNA levels. Thus, Rom2 expression level is regulated at both the mRNA and protein levels. How do Khd1 and Ccr4 positively regulate the expression of *ROM2*? The myc-tagged *ROM2* construct used in Fig. 4A and 5 had the *ADH1* 3′ UTR instead of the endogenous *ROM2* 3′ UTR, and the Rom2myc protein levels were decreased in *ccr4Δ* single mutant and *khd1Δ ccr4Δ* double mutant cells, implying that the *ROM2* 3′ UTR seems not to be essential for the regulation of *ROM2* expression. In the case of the regulation of *MTL1* mRNA stability by Khd1, *MTL1* mRNA itself bears the multiple CNN repeats involved in destabilization by the decapping enzyme Dcp1/2 and the 5′-to-3′ exonuclease Xrn1, and Khd1 stabilizes *MTL1* mRNA by binding to this element (23, 24). Since *ROM2* mRNA contains three CNN repeats in the coding sequence and Khd1 associates with *ROM2* mRNA (11, 23), Khd1, together with Ccr4, may stabilize *ROM2* mRNA by binding to the CNN repeats of the *ROM2* mRNA. Since Rom2 expression is regulated at both mRNA and protein levels, the binding to the CNN repeats by Khd1 may also be involved in translational control. Consistently, the HA-Rom2 protein levels expressed from the *GAL1* promoter were decreased in *ccr4Δ* and *khd1Δ ccr4Δ* mutant cells compared to wild-type cells, while the *HA-ROM2* mRNA levels were not altered. Additionally, Mpk1, which is activated in *ccr4Δ* and *khd1Δ ccr4Δ* mutants, may be involved in the decrease of the *ROM2* mRNA. The decreased Rom2myc protein levels in *ccr4Δ* and *khd1Δ ccr4Δ* mutant cells were partially suppressed by the *mpk1Δ* mutation, implying the possibility that Mpk1 is also involved in *ROM2* expression at the protein level.

While the *ccr4Δ* mutant displays a synthetic growth defect with the *khd1Δ* mutation, the *dhh1Δ* mutant does not. The simple explanation is that in the *ccr4Δ* mutant, where Rom2 function is decreased and Lrg1 function is increased, Rho1 activity is severely decreased. Consistently, a constitutively active *RHO1* allele is able to suppress the growth defect of the *khd1Δ ccr4Δ* double mutant (11) (Fig. 12A). In the *dhh1Δ* mutant, where Rom2 function is normal and Lrg1 function is increased, the decrease in Rho1 activity is lower than that in the *ccr4Δ* mutant. This raises the possibility that *khd1Δ* mutation would affect cell growth only when Rho1 activity is more severely impaired. However, this explanation is not consistent with the *pop2Δ* case. The *pop2Δ* mutant displays a synthetic growth defect with the *khd1Δ* mutation, but Rom2 function is normal in the *pop2Δ* mutant. In the *pop2Δ* mutant, where Rom2 function is normal and Lrg1 function is increased, the decrease in Rho1 activity is lower than that in the *ccr4Δ* mutant. Since a constitutively active *RHO1* allele cannot suppress the growth defect of the *khd1Δ pop2Δ* double mutant (Fig. 12B), the *khd1Δ pop2Δ* double mutant might have an additional defect in the CWI signaling pathway. Intriguingly, the growth defect of the *khd1Δ pop2Δ* double mutant as well as the *khd1Δ ccr4Δ* double mutant could be suppressed by the constitutively active *PKC1* allele. These results suggest that Pop2 and Ccr4 not only destabilize a common target, *LRG1* mRNA, but also regulate the CWI pathway at different points, and that Ccr4 and Pop2 act at a point upstream of Pkc1 in the CWI pathway. Although Rho1 activity is severely decreased in the *khd1Δ ccr4Δ* double mutant, where Rom2 function is decreased and Lrg1 function is increased, Mpk1 seems to be activated in the *khd1Δ ccr4Δ* double mutant. Since Lrg1 participates in the regulation of β-1,3-glucan synthase (16), one possibility is that the decreased Rho1 activity could not activate β-1,3-glucan synthase due to the increased Lrg1 but could still activate the Pkc1-Mpk1 branch in the *khd1Δ ccr4Δ* double mutant. The levels of expression of *BEM2* and *SAC7*, which are involved in the downregulation of the Pkc1-activated MAPK pathway (17, 18), were not altered significantly in the *khd1Δ ccr4Δ* double mutant (data not shown). Regulation of the levels of different Rho1-GAPs by modulation of mRNAs might ensure Rho1 activation in a target-specific manner.

Previously, we revealed that Ccr4, a component of the Ccr4-Not cytoplasmic deadenylase complex, functions in the CWI pathway (11). In this study, we further identified Pop2 deadenylase and Dhh1 DExD/H box protein as the regulator of the CWI pathway. Ccr4, Pop2, and Dhh1 modulate the levels of mRNAs for specific Rho1 regulators, Rom2 and Lrg1. In budding yeast, Rho1 activity is tightly regulated both temporally and spatially (12). It is anticipated that Ccr4, Pop2, and Dhh1 may contribute to the precise spatiotemporal control of Rho1 activity by regulating expression of its regulators temporally and spatially. Therefore, to further elucidate how Ccr4, Pop2, and Dhh1 regulate *ROM2* and *LRG1* mRNAs will undoubtedly provide valuable insights into the precise spatiotemporal regulation of this signaling pathway.

## MATERIALS AND METHODS

### Strains and general methods.

*Escherichia coli* DH5α was used for DNA manipulations. *S*. *cerevisiae* strains used in this study are described in Table 1. Standard procedures were followed for yeast manipulations (25). The media used in this study included rich medium, synthetic complete medium (SC), and synthetic minimal medium (SD) (25). SC lacking amino acids or other nutrients (e.g., SC−Ura is SC lacking uracil) were used to select transformants. Recombinant DNA procedures were carried out as described previously (26).

**TABLE 1:** *S*. *cerevisiae* strains used in this study

Strain	Genotype	Reference
10B	*MATα ade2 trp1 can1 leu2 his3 ura3 GAL psi^+^ HOp-ADE2-HO 3′ UTR*	33
10BD	*MAT****a***/*MATα ade2*/*ade2 trp1*/*trp1 can1*/*can1 leu2*/*leu2 his3*/*his3 ura3*/*ura3*	33
10BD-c163	*MAT****a***/*MATα ade2*/*ade2 trp1*/*trp1 can1*/*can1 leu2*/*leu2 his3*/*his3 ura3*/*ura3 KHD1*/*khd1Δ*::*CgHIS3* *CCR4*/*ccr4Δ*::*CgLEU2*	11
10BD-p163	*MAT****a***/*MATα ade2*/*ade2 trp1*/*trp1 can1*/*can1 leu2*/*leu2 his3*/*his3 ura3*/*ura3 KHD1*/*khd1Δ*::*CgHIS3* *POP2*/*pop2Δ*::*CgLEU2*	11
10BD-d163	*MAT****a***/*MATα ade2*/*ade2 trp1*/*trp1 can1*/*can1 leu2*/*leu2 his3*/*his3 ura3*/*ura3 KHD1*/*khd1Δ*::*CgHIS3* *DHH1*/*dhh1Δ*::*CgLEU2*	11
c1H-1A	*MATα ade2 trp1 can1 leu2 his3 ura3*	11
c1H-1B	*MATα ade2 trp1 can1 leu2 his3 ura3 khd1Δ*::*CgHIS3 ccr4Δ*::*CgLEU2*	11
c1H-1C	*MAT**a** ade2 trp1 can1 leu2 his3 ura3 khd1Δ*::*CgHIS3*	11
c1H-1D	*MAT**a** ade2 trp1 can1 leu2 his3 ura3 ccr4Δ*::*CgLEU2*	11
p1H-2A	*MATα ade2 trp1 can1 leu2 his3 ura3*	This study
p1H-2B	*MATα ade2 trp1 can1 leu2 his3 ura3 pop2Δ*::*CgLEU2*	This study
p1H-2C	*MAT**a** ade2 trp1 can1 leu2 his3 ura3 khd1Δ*::*CgHIS3 pop2Δ*::*CgLEU2*	This study
p1H-2D	*MAT**a** ade2 trp1 can1 leu2 his3 ura3 khd1Δ*::*CgHIS3*	This study
d1H-1A	*MATα ade2 trp1 can1 leu2 his3 ura3*	This study
d1H-1B	*MATα ade2 trp1 can1 leu2 his3 ura3 khd1Δ*::*CgHIS3 dhh1Δ*::*CgLEU2*	This study
d1H-1C	*MAT**a** ade2 trp1 can1 leu2 his3 ura3 khd1Δ*::*CgHIS3*	This study
d1H-1D	*MAT**a** ade2 trp1 can1 leu2 his3 ura3 dhh1Δ*::*CgLEU2*	This study
10BD-c163-r1	*MAT****a***/*MATα ade2*/*ade2 trp1*/*trp1 can1*/*can1 leu2*/*leu2 his3*/*his3 ura3*/*ura3 KHD1*/*khd1Δ*::*CgTRP1* *CCR4*/*ccr4Δ*::*CgLEU2 ROM1*/*rom1Δ*::*CgHIS3*	11
10BD-p163-r1	*MAT****a***/*MATα ade2*/*ade2 trp1*/*trp1 can1*/*can1 leu2*/*leu2 his3*/*his3 ura3*/*ura3 KHD1*/*khd1Δ*::*CgTRP1* *POP2*/*pop2Δ*::*CgLEU2 ROM1*/*rom1Δ*::*CgHIS3*	This study
10BD-d1-r1	*MAT****a***/*MATα ade2*/*ade2 trp1*/*trp1 can1*/*can1 leu2*/*leu2 his3*/*his3 ura3*/*ura3 DHH1*/*dhh1Δ*::*CgLEU2* *ROM1*/*rom1Δ*::*CgHIS3*	This study
180-3B-4A	*MATα ade2 trp1 can1 leu2 his3 ura3 ROM2myc-kan*	This study
180-3B-1B	*MATα ade2 trp1 can1 leu2 his3 ura3 khd1Δ*::*CgHIS3 ccr4Δ*::*CgLEU2 ROM2myc-kan*	This study
180-3B-7D	*MAT**a** ade2 trp1 can1 leu2 his3 ura3 ccr4Δ*::*CgLEU2 ROM2myc-kan*	This study
180-3B-7C	*MAT**a** ade2 trp1 can1 leu2 his3 ura3 khd1Δ*::*CgHIS3 ROM2myc-kan*	This study
180-m-1D	*MATα ade2 trp1 can1 leu2 his3 ura3 ROM2myc-kan*	This study
180-m-3D	*MATα ade2 trp1 can1 leu2 his3 ura3 khd1Δ*::*CgHIS3 ccr4Δ*::*CgLEU2 ROM2myc-kan*	This study
180-m-7A	*MAT**a** ade2 trp1 can1 leu2 his3 ura3 khd1Δ*::*CgHIS3 ccr4Δ*::*CgLEU2 mpk1Δ*::*CgHIS3 ROM2myc-kan*	This study
180-m-6B	*MAT**a** ade2 trp1 can1 leu2 his3 ura3 ccr4Δ*::*CgLEU2 ROM2myc-kan*	This study
180-m-6C	*MAT**a** ade2 trp1 can1 leu2 his3 ura3 ccr4Δ*::*CgLEU2 mpk1Δ*::*CgHIS3 ROM2myc-kan*	This study
cl4-1B	*MATa ade2 trp1 can1 leu2 his3 ura3 ccr4Δ*::*CgLEU2 lrg1Δ*::*CgHIS3*	This study
pl4-1B	*MATa ade2 trp1 can1 leu2 his3 ura3 pop2Δ*::*CgLEU2 lrg1Δ*::*CgHIS3*	This study
dl4-1B	*MATa ade2 trp1 can1 leu2 his3 ura3 dhh1Δ*::*CgLEU2 lrg1Δ*::*CgHIS3*	
10BD-c163-r2l1	*MAT****a***/*MAT*α *ade2*/*ade2 trp1*/*trp1 can1*/*can1 leu2*/*leu2 his3*/*his3 ura3*/*ura3 KHD1*/*khd1Δ*::*CgTRP1* *CCR4*/*ccr4Δ*::*CgLEU2 ROM2*/*rom2Δ*::*CgHIS3 LRG1*/*lrg1Δ*::*KlURA3*	This study
10BD-p-r2l1	*MAT****a***/*MATα ade2*/*ade2 trp1*/*trp1 can1*/*can1 leu2*/*leu2 his3*/*his3 ura3*/*ura3 POP2*/*pop2Δ*::*CgLEU2* *ROM2*/*rom2Δ*::*CgHIS3 LRG1*/*lrg1Δ*::*KlURA3*	This study
10BD-p-r2l1	*MAT****a***/*MATα ade2*/*ade2 trp1*/*trp1 can1*/*can1 leu2*/*leu2 his3*/*his3 ura3*/*ura3* *DHH1*/*dhh1Δ*::*CgLEU2ROM2*/*rom2Δ*::*CgHIS3 LRG1*/*lrg1Δ*::*KlURA3*	This study
10BD-c163-d1l1	*MAT****a***/*MATα ade2*/*ade2 trp1*/*trp1 can1*/*can1 leu2*/*leu2 his3*/*his3 ura3*/*ura3 KHD1*/*khd1Δ*::*CgTRP1* *CCR4*/*ccr4Δ*::*CgLEU2 DHH1*/*dhh1Δ*::*CgHIS3 LRG1*/*lrg1Δ*::*KlURA3*	This study

### Plasmids.

Plasmids used in this study are described in Table 2. Plasmids pCgLEU2, pCgHIS3, and pCgTRP1 are pUC19 carrying the *Candida glabrata* *LEU2*, *HIS3*, and *TRP1* genes, respectively (27). Plasmid pKlURA3 is pUC19 carrying the *Kluyveromyces lactis URA3*. Plasmid pGAL-HA-LRG1 expressing *HA-LRG1* from the *GAL1* promoter was used for the experiment for *LRG1* mRNA degradation. Plasmid YCplac33-ROM2myc expressing *ROM2myc* from the endogenous promoter and plasmid pGAL-HA-ROM2 expressing *HA-ROM2* from the *GAL1* promoter were used for Western blotting of Rom2 protein.

**TABLE 2:** Plasmids used in this study

Plasmid	Relevant marker(s)	Reference
YCplac33	*URA3 CEN-ARS*	34
pRS316-GAL-LRG1	*URA3 CEN-ARS* pGAL-LRG1-LRG1 3′ UTR	This study
YCplac33-ROM2myc	*URA3 CEN-ARS* pROM2-ROM2myc-ADH1 3′ UTR	This study
pRS316-GAL-HA-ROM2	*URA3 CEN-ARS* pGAL-HA-ROM2-ROM2 3′ UTR	This study
YCplac33-RHO1-Q86L	*URA3 CEN-ARS RHO1-Q86L*	35
pRS316-PKC1-R398P	*URA3 CEN-ARS PKC1-R398P*	36
YEplac195	*URA3* 2μ	34
YEplac195-POP2	*URA3* 2μ *POP2*	7
YEplac195-DHH1	*URA3* 2μ *DHH1*	7
YEplac195-CCR4	*URA3* 2μ *CCR4*	7
YEplac195-ROM2	*URA3* 2μ *ROM2*	11
pCgLEU2	*C. glabrata* LEU2 in pUC19	27
pCgHIS3	*C. glabrata* HIS3 in pUC19	27
pCgTRP1	*C. glabrata* TRP1 in pUC19	27
pKlURA3	*K. lactis* URA3 in pUC19	11
pKlURA3	*K. lactis* URA3 in pUC19	11
pFA6a-13myc-kanMX6	myc	30

### Gene deletion and protein tagging.

Deletions of *KHD1*, *CCR4*, *POP2*, *DHH1*, *ROM1*, *ROM2*, and *LRG1* were constructed by PCR-based gene deletion method (27–29). Primer sets were designed such that 46 bases at the 5′ ends of the primers were complementary to those at the corresponding region of the target gene and 20 bases at their 3′ ends were complementary to the pUC19 sequence outside the polylinker region in the plasmid pCgLEU2, pCgHIS3, pCgTRP1, or pKlURA3. Primer sets for PCR were designed to delete the open reading frame (ORF) completely. The PCR products were transformed into the wild-type strain and selected for Leu^+^, His^+^, Trp^+^, or Ura^+^. The *ROM2myc* strains were prepared by the method of Longtine et al. (30) using pFA6a-13myc-kanMX6.

### Northern blot analysis.

Total RNA was prepared from cells using Isogen reagent (Nippon Gene) and RNeasy minikit (Qiagen). RNA samples were separated by 1.5% denatured agarose gel electrophoresis and transferred to a nylon membrane. Then, RNA was hybridized using digoxigenin (DIG)-labeled antisense probe. The primer pair j298 (TGACGATATGATGAGCTCCTCCTTACGTCA) and j297 (TTAACCCCAGAAATCTAACGACG) and primer pair j259 (ATGATTCAAAATTCTGCTGGTTA) and j260 (GCCAATATTTATGAATTCCATAAC) were used to detect transcript containing *ROM2* and *LRG1*, respectively. After washing and blocking, the membrane was incubated with alkaline phosphatase-conjugated anti-DIG antibody, and the signal was detected by enhanced chemiluminescence.

mRNA degradation was determined from Northern blots as described previously (31, 32). Cells were grown in SG−Ura, and the medium was changed to SC−Ura to inhibit transcription from the *GAL1* promoter. Cells were harvested at the times indicated in the figures, and total RNA was isolated. Samples were analyzed by Northern blotting with specific probes, and half-lives (*t*_1/2_) (in minutes) were determined as the means from three independent experiments.

### Western blot analysis.

Extracts were prepared as described previously (23, 24, 33). Extracts were subjected to SDS-PAGE on 8% acrylamide gels followed by electroblotting onto an Immobilon membrane (Millipore). To detect myc-tagged and hemagglutinin (HA)-tagged proteins, the membrane was incubated with anti-myc antibody (9E10; Santa Cruz Biotechnology) (1:2,000) and anti-HA antibody (HA11; Santa Cruz Biotechnology) (1:2,000), respectively, and then with HRP-labeled secondary antibody (Calbiochem) (1:4,000). To control for equal loading of the lanes, the blots were probed with anti-Mcm2 antibody (Santa Cruz Biotechnology) (1:1,000) or anti-Pgk1 antibody (Invitrogen) (1:1,000) and peroxidase-conjugated secondary antibody (Calbiochem) (1:3,000).
